# Probable Locally Acquired *Babesia divergens–*Like Infection in Woman, Michigan, USA

**DOI:** 10.3201/eid2408.180309

**Published:** 2018-08

**Authors:** Erica Herc, Bobbi Pritt, Taylor Huizenga, Richard Douce, Matthew Hysell, Duane Newton, Jennifer Sidge, Eve Losman, John Sherbeck, Daniel R. Kaul

**Affiliations:** Henry Ford Hospital, Detroit, Michigan, USA (E. Herc);; Mayo Clinic, Rochester, Minnesota, USA (B. Pritt);; Michigan State College of Osteopathic Medicine, East Lansing, Michigan, USA (T. Huizenga);; Lakeland Healthcare, Saint Joseph, Michigan, USA (R. Douce, M. Hysell);; University of Michigan Medical School, Ann Arbor, Michigan, USA (D. Newton, E. Losman, J. Sherbeck, D.R. Kaul);; Michigan Department of Health and Human Services, Lansing, Michigan, USA (J. Sidge)

**Keywords:** Babesia divergens, B. divergens–like/MO-1, parasites, protozoa, babesiosis, emerging infection, asplenic patient, tickborne disease, ticks, vector-borne infections, erythrocyte exchange transfusion, parasitemia, Michigan, United States

## Abstract

We report an asplenic patient who was infected with *Babesia divergens*–like/MO-1. The clinical course was complicated by multiorgan failure that required intubation and dialysis. The patient recovered after an exchange transfusion and antimicrobial drug therapy. Physicians should be alert for additional cases, particularly in asplenic persons.

Babesiosis is an emerging threat in North America. In 2014, this disease was reported in 31 states in the United States (*1*). Protozoan intraerythrocytic parasites of the genus *Babesia* cause infection when transmitted by ticks or blood transfusions. Infections occur most frequently in spring or early summer, coinciding with the host-seeking activity of *Ixodes scapularis* nymphal ticks. Most cases occur in the northeastern upper midwestern United States. Most infections in the United States are caused by *B. microti*, which was first identified in 1966. Other *Babesia* species, including *B. duncani* and *B. divergens*–like/MO-1, have been rarely reported (*2*).

Clinical manifestations of babesiosis can range from asymptomatic to multiorgan failure. Severe illness is frequently seen in elderly, immunocompromised, and asplenic patients (*3*). We report a case of severe babesiosis caused by a *B. divergens*–like/MO-1 organism in an asplenic woman. This case was probably acquired in western Michigan.

## The Study

A 60-year-old woman with hereditary spherocytosis status postsplenectomy and a history of pancreatic and colon cancer status post-Whipple procedure was hospitalized in 2017 because she had multiorgan failure. Fatigue, nausea, dyspnea, weakness, and chest pressure without fever had developed 5 days earlier. She was tachycardic and had jaundice but had otherwise reference (normal) vital signs. 

Results of testing in the emergency department showed a leukocyte count of 20,800 cells/μL, hemoglobin 8.5 g/dL (reference 10.5 g/dL), creatinine of 5.3 mg/dL, lactate dehydrogenase 7,340 U/L, and haptoglobin <10 mg/dL, and increased levels of liver enzymes (aspartate aminotransferase 128 U/L, alanine aminotransferase 43 U/L, and total bilirubin 9.7 mg/dL). Peripheral blood smear results showed numerous intraerythrocytic parasites consistent with a *Babesia* sp.; parasitemia was 25%–30% (Figure, panel A).

**Figure:**
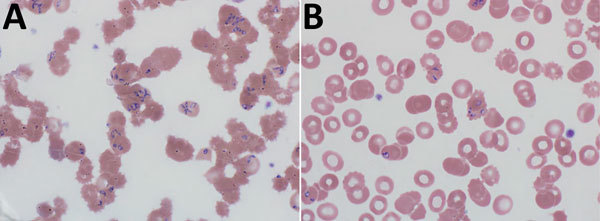
Peripheral blood films for a 60-year-old woman with probable locally acquired *Babesia divergens–*like infection, Michigan, USA. A) Before erythrocyte exchange transfusion. Parasitemia was 25%–30%. B) After erythrocyte exchange transfusion. Parasitemia was 3.5%. Original magnification ×1,000.

Given her multiorgan failure, the patient was transferred to a tertiary care center for exchange transfusion. At transfer, she was delirious and was admitted to the intensive care unit. She was given quinidine and clindamycin and underwent a 2-volume erythrocyte exchange transfusion. After exchange transfusion, parasitemia decreased to 3.5% (Figure, panel B). The following day she was given clindamycin (600 mg every 8 h), atovaquone (750 mg 2×/d), and azithromycin (250 mg/d) because of prolonged QTc.

Multiplex real-time PCR specific for a 204-bp region of the 18S rDNA gene (*4*) performed at a reference laboratory was positive for *B. divergens*–like/MO-1 and negative for *B. microti* and *B. duncani*. This result was confirmed by additional PCR testing at the Centers for Disease Control and Prevention (Atlanta, GA, USA). Serologic testing results were negative for *Borrelia burgdorferi* and *Anaplasma phagocytophilum* antibodies. No specific serologic analysis was performed for *B. microti* or *B. duncani*.

The patient required mechanical ventilation, pressor support, and renal replacement therapy. Serial peripheral blood smears showed the following consecutive parasitemia values over a 13-day period: 25%–30%, 3.5%, 2%, 1.8%, 0.5%, <0.1%, and 0%. Her hemoglobin and platelet levels returned to reference ranges during this period. Seven days after admission, she was extubated and renal function eventually improved. Antimicrobial drugs were continued after discharge for 4 weeks. At follow-up on day 29 postpresentation after her initial emergency department visit, her clinical status continued to improve, and repeat peripheral blood smears were negative for *Babesia* spp.

The patient gardened at her home in Berrien County, Michigan, USA, and walked along the Lake Michigan shoreline. She did not report any known tick bites, but the region is known to be endemic for *Ixodes scapularis* ticks. She had traveled to Kansas City, Missouri, USA, 2.5 weeks before symptom onset, but stayed in urban nonpark areas and did not have contact with animals. She did not have blood transfusions during the year before her illness. Given this history, the most likely source of disease acquisition was in Michigan.

## Conclusions

We report a case of severe *B. divergens*–like/MO-1 infection in the upper midwestern United States. The patient probably contracted the disease from a tick in southwestern Michigan. She did not have any blood transfusions within the previous year, and the longest reported period between transfusion-transmitted babesiosis and a recipient diagnosis is 384 days (*5*).

Although *B. microti* is the predominant cause of babesiosis in the United States, 5 cases caused by *B. divergens*–like organisms have been reported. Reports include residents of Missouri (1992 and 2010), Kentucky (2001), Washington (2002), and Arkansas (2017) (*4*,*6*–*8*). All case-patients were asplenic and had high levels of parasitemia. Three of these 5 patients died. Neither of the survivors received an exchange transfusion. Of the patients who died, 1 received an exchange transfusion, 1 did not, and the status for the third patient was unknown. *B.* *divergens*–like/MO-1 parasites have also been identified in cottontail rabbits and *Ixodes* spp. ticks on Nantucket Island, Massachusetts, USA (*9*).

In Europe, *B. divergens* is the most frequent cause of human babesiosis (≈40 reported cases), although the seroprevalence might be higher; 13% of patients with Lyme disease were seropositive in Sweden (*10*,*11*). Further analysis of cases of *B. divergens*–like/MO-1 infection in the United States showed that this infection is distinct from that of *B. divergens* in Europe on the basis of sequence analysis, lack of infectiousness to cattle, and distinct morphologic differences when grown in vitro (*12*). A case of *B. divergens*–like infection was reported in a patient on the Canary Islands in 1994 (*11*). Sequence analysis showed similarities to *B. divergens* but neither the organism nor its vector was present on the islands. Two possible *B. divergens*–like species were also identified in China in 2011 (*11*).

The distribution and number of babesiosis infections in the United States is increasing. In 2014, babesiosis was reported in 31 states, compared with 27 in 2013. The number of reported cases increased from 1,126 in 2011 to nearly 1,744 in 2014. Seroprevalance data from disease-endemic regions ranged from 6% to 16% for *B. microti*, which suggests the reported disease prevalence is underestimated (*2*). In 2016, there were 2 confirmed cases of *B. microti* infection in residents of Michigan, both of whom lived near the Wisconsin border. However, *B. microti* was not detected in the tick vector.

Although microscopic features are similar for all human-infecting *Babesia* species, *B. divergens* and *B. divergens*–like organisms are more likely to have tetrad forms (Maltese cross forms) and accole forms on peripheral blood smears than *B. microti*. Octad forms are rarely seen.

Treatment recommendations for *Babesia* infections are made on the basis of data for *B. microti* because clinical information regarding B. *divergens–*like infections is limited to case reports and treatment recommendations are available elsewhere (*3*). A recent study supports treating persons who are immunosuppressed for >6 weeks, including persons with negative blood smears for 2 weeks before discontinuation of therapy (*13*). In case-patients with parasitemia >10% or evidence of end-organ dysfunction, an emergent automated erythrocyte exchange transfusion is indicated. A 2-volume erythrocyte exchange should lead to a 90% reduction of parasite load (*14**,**15*). This procedure not only removes parasite-infected erythrocytes but also removes vasoactive factors, including thromboplastic substances and cytokines, which contribute to development of disseminated intravascular coagulation and renal failure (*14*).

In summary, babesiosis is a potentially fatal disease caused by protozoan parasites of the genus *Babesia*. *B. divergens–*like/MO-1 infections are rare in the United States, and many patients who are infected had major illness and high mortality rates. Physicians should be alert for additional cases, particularly in asplenic persons. Further epidemiologic investigations of ticks in the upper midwestern United States for *B. divergens*–like organisms are warranted.
